# The Role of User Input in Shaping Online Information From the National Cancer Institute

**DOI:** 10.2196/jmir.7.3.e25

**Published:** 2005-07-01

**Authors:** Lakshmi M Grama, Margaret Beckwith, Wayne Bittinger, Diana Blais, Cindy Lollar, Anne Middleswarth, Marianne Noone, Deborah Price, Sharon Quint-Kasner, Victoria Shields, Lawrence W Wright

**Affiliations:** ^1^Office of Cancer Information Products and SystemsOffice of CommunicationsNational Cancer InstituteNational Institutes of HealthDepartment of Health and Human ServicesBethesda, MDUSA

**Keywords:** Cancer information, Internet, online information, usability, website

## Abstract

The National Cancer Institute (NCI) was among the first federal agencies to recognize the potential of the Internet for disseminating health-related information. The evolution and refinement of NCI's online cancer information has been substantially “user driven”—from the launch of CancerNet in 1995 to the recent redesign of its award-winning successor, the NCI website. This article presents an overview of NCI's multi-pronged approach to gathering input about its online information products, including stakeholder meetings, focus groups, standard and customized online user surveys, usability testing, heuristic reviews, and search log analysis. Also highlighted are some of the many enhancements that have been made to NCI's online cancer information products based on user input.

## Introduction

The National Cancer Institute (NCI) was among the first federal agencies to recognize the potential of the Internet for disseminating health-related information, and it launched its CancerNet website in 1995. This site was a natural extension of NCI's information dissemination efforts, which have been carried out in response to mandates from Congress in the National Cancer Act of 1971 [1] and subsequent legislation. Table 1 outlines major milestones in the development of NCI's Web presence.

**Table 1:** Milestones in the development of NCI's website

**Year**	**Milestone**
1995	CancerNet website is launched.
1999	cancerTrials website is launched.
1999	CancerNet website is redesigned.
2002	NCI's overarching website [2] is redesigned; CancerNet and cancerTrials websites are subsumed into the redesigned site.
2004	NCI website is redesigned.

A large part of NCI's pre-1995 information dissemination efforts was targeted at health professionals through the Physician Data Query (PDQ^®^) cancer information database, which contains information summaries on numerous cancer-related topics and a cancer clinical trials registry. PDQ was available to medical librarians, physicians, oncology nurses, and other professionals through the National Library of Medicine's online information system [3-6].

There was, however, a new dynamic in the development of the Web. Cancer patients were coming online in large numbers, seeking to be informed decision makers in their own care. Simultaneously, the patient advocacy community was becoming more vocal in requesting that NCI provide products geared to patients. NCI responded to this growing audience by organizing the CancerNet website by audience type, with entry points for patients, health professionals, and researchers, and with information categorized accordingly.

The evolution and refinement of NCI's online cancer information has been notably “user driven.” NCI has adopted a multifaceted approach to gathering feedback and other information about how its information products are used. This has included pre- and post-design tests in usability labs, heuristic or expert review, informal user feedback, standard online user surveys, focus groups, analysis of site usage and search logs, and special user survey projects. Each generation of NCI's Web presence has been informed by user feedback. NCI staff members were crucial leaders in developing usability guidelines and standards that are now widely accepted in the industry, and NCI was one of the first federal agencies to conduct systematic usability testing with its CancerNet website.

This article presents an overview of the methods NCI has used to gather input about its online information products and services. It is not the result of research projects that set out to test specific hypotheses about the impact of specific user-driven enhancements. Rather, it presents an approach to information architecture and design of a website that uses a variety of methods to gauge user behaviors and preferences. It highlights some of the many enhancements that have been implemented in response to user data and feedback. While NCI's website contains a wealth of additional information about cancer research opportunities, funding, NCI programs/initiatives, cancer statistics, and information for the news media, space limitations prevent a discussion of the role of user input in the design and implementation of these areas. The focus of this article will be enhancements to patient-oriented cancer information and information about clinical trials.

## How NCI Gathers User Input and Feedback

### Stakeholder Input

NCI solicits user input prior to any major online system design or redesign. For example, in response to a growing need for clinical trials information, and prior to a major redesign of the backend database and the user interface of its CancerNet website, NCI organized the Clinical Trials Information System meeting in Chantilly, Virginia, USA, in 1998.

Approximately 200 patients, advocates, clinicians, oncology nurses, clinical investigators, and health information providers representing the core users of NCI's online information resources came together to brainstorm the design of a clinical trials information system.

**Figure 1:**
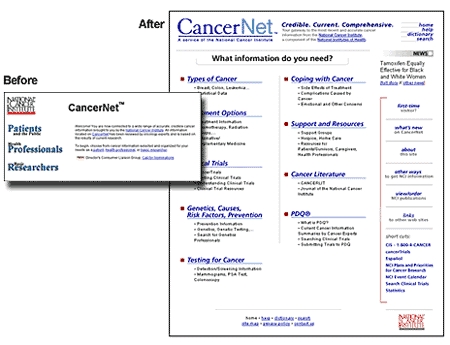
Before and after screen shots of the CancerNet home page, showing a shift in focus from audience to topic with the 1999 redesign

Some of the key recommendations of the meeting were the following: (1) that the NCI website avoid segmenting information pathways according to type of user (patient, physician, researcher); (2) that information be customized to provide varying degrees of technical detail, complexity, and reading level; and (3) that users be able to easily move between these levels. It was also recommended that the NCI website integrate clinical trials information with the full spectrum of cancer information; include information about clinical trials, patient rights, and the informed consent process; and include a feature covering news topics related to clinical trials.

One of the outcomes of this meeting was the development of a new NCI website, cancerTrials, to provide an educational context for the PDQ clinical trials registry that was offered on CancerNet. The cancerTrials website was launched in 1999. In addition to guidance on how to search the PDQ registry, visitors to the new site were offered original articles explaining what cancer clinical trials were, how they worked, and where to find them. They also were offered brief summaries of recently announced cancer trial results and other timely news related to the US clinical trials system.

The subsequent redesign of CancerNet in 1999 [7] carried out the Chantilly recommendation to abandon the partition of the site by audience (Figure 1). Now, the site gave all users information organized around a standard set of topics. Information was presented at varying levels for most of the common cancers—including the “What You Need To Know” series for the most basic introduction, patient and health professional versions of the PDQ cancer information summaries, and abstracts (summaries) of clinical trial protocols written for patients and health professionals. The new design also made it easy for users to switch between the different information levels. Input obtained at the Chantilly meeting continues to influence the development of NCI's cancer information products and their presentation to users.

### Ongoing Feedback from CIS Information Specialists

Information specialists at NCI's Cancer Information Service (CIS) are the front line of NCI's interactions with the cancer community, particularly the public [8]. Through the CIS toll-free telephone service (1-800-4-CANCER) and “LiveHelp” online chat sessions, information specialists help individuals who are seeking cancer information. As needed, they can assist callers and website visitors with NCI online tools and resources. As “power users” of the NCI website, they often help test new features. Regular feedback from the CIS to website staff helps drive website improvements.

### User Surveys

A critical factor in achieving continuous improvement of NCI's Web resources is soliciting user feedback to learn what works, what doesn't, and where gaps in information or functionality exist. In preparation for the 1999 redesign of CancerNet, an online survey asked users to identify the information they were seeking (Table 2), difficulties they encountered on the site, features they found useful, and additional information or features that were needed. Users were also asked about their general Web usage and basic demographics.

**Table 2:** Type of information users were seeking on CancerNet (1999)

**Percentageof Respondents(N = 780)**	**Information Sought**
22.8	Information on a specific type of cancer
18.6	Treatment information (general and specific)
11.3	Clinical trial information (specific trials, general information, trial results)
8.1	Symptoms of cancer, causes, risk factors, detection, diagnosis, prevention
6.8	Specific term (type of tumor or other term—not by name of cancer)
4.7	Cancer literature/articles
4.5	New treatments, news, recent findings, current research projects
3.5	Patient support (pain relief, diet/nutrition, survivorship, exercise, follow-up, questions to ask doctor)
3.1	Side effects
2.9	Statistics (incidence rates, survival rates, mortality rates)
2.8	Drug information
2.8	Access to other cancer resources (treatment facilities, physician names/specialties, national tumor registry, cost information, insurance coverage, patient support group)
2.2	Caregiver information (how to help patient, what to expect as disease progresses, how to talk to patient, etc)
2.1	History of cancer research, information for reports/projects
1.8	NCI publications (ordering information)
0.8	Alternative treatments
0.6	Genetic information (general and specific)
0.4	Search engine for the site
0.3	Information about oncology professions

**Figure 2:**
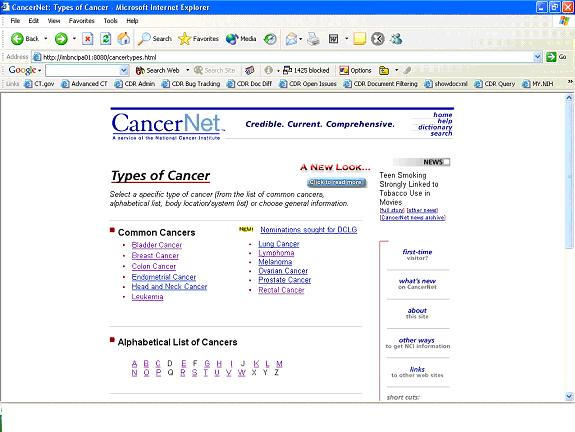
The “Types of Cancer” page on CancerNet

**Figure 3:**
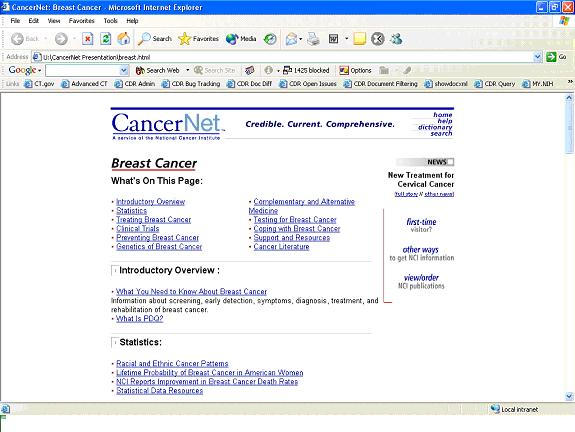
The “Breast Cancer” page on CancerNet, an example of a cancer-specific home page

Feedback from the online survey, along with input from the Chantilly meeting, guided the redesign of CancerNet in 1999. On the redesigned site, users could start with the “Types of Cancer” page (Figure 2), which enabled users to quickly find information about specific cancers. They could then choose a cancer-specific home page (Figure 3), where information related to the cancer was organized by topics such as “Introductory Overview,” “Statistics,” “Treatment,” and “Clinical Trials.”

In 2002, NCI's overarching website was redesigned, and the CancerNet and cancerTrials websites became the Cancer Information and Clinical Trials portal areas of the redesigned site. In 2004, the NCI site underwent another redesign, once again guided by extensive evaluation and user input.

NCI's early decision to provide information tailored for patients and their families continues to be supported by surveys conducted during the past five years. Data from 1999 showed that 44% of visitors to the site described themselves as cancer patients or family members or friends of a cancer patient. Data from the American Customer Satisfaction Index (ACSI) survey posted on the NCI website in 2004 showed that more than 50% of respondents identified themselves as cancer patients or family or friends. The next largest audience in 2004 was health care providers, about 13%. NCI continues to keep the patient at the center of many of its online resources—PDQ's cancer information summaries and clinical trial abstracts, clinical trial results summaries, fact sheets and other information products, and the website's dictionary are all written for lay audiences.

### American Customer Satisfaction Index (ACSI Survey)

Both before and after the 2004 redesign, the website displayed the ACSI survey [9]. This survey gathers input from users at points within the website. The ACSI survey can be utilized site-wide or for a certain URL. One version of the survey can be posted to appear randomly on all pages of the site (Appendix 1), and another can be set to appear on a group of related pages to collect in-depth data on a particular subject.

The ACSI methodology provides continuous online feedback and is a uniform, national, cross-industry measure of customer satisfaction. A core set of ACSI questions measures overall satisfaction, and customized questions can be added regarding individual websites or pages.

Data from the survey are helpful in supporting or dispelling impressions of who uses a site and what their information needs are. For example, data from the 2004 ACSI survey showed that approximately 57% of NCI's website visitors are first-time users of the site. This underscores the need for intuitive site structure and navigation tools that can be easily grasped by users with no prior knowledge of the site. Multiple paths to core information, such as cancer-specific home pages and clinical trial search tools, were created in 2004 to help new users easily find the most sought-after information. While we cannot make a direct correlation between these enhancements and increased customer satisfaction, the ACSI survey results published in December 2004 named the NCI website the “best in customer satisfaction” in the portal/department main site category [10]. Overall satisfaction among visitors to major government online portals was 72.1, on a scale of 0 to 100. The NCI website led the category for government sites with an overall satisfaction score of 80. In the first quarter of 2005, the NCI website was again the highest scoring government portal site, with a score of 80. NCI expects to further analyze ACSI data to inform additional improvements to the website.

### Usability Testing and Expert Review

Usability testing helps ensure that products and services address the needs and interests of website visitors [11,12]. In lab sessions with representative users, testers pose scenarios (see Appendix 2) and solicit comments to gauge the effectiveness of page designs, functions, navigation paths, labels and terminology, and other elements. Data from iterative testing inform the refinement of key pages and the development of new features. NCI also consults with experts on user-centered design to help ensure that its information products keep pace with current standards and trends. For example, prior to the launch of the redesigned NCI website in 2004, a panel of experts was involved in heuristic reviews, and their recommendations led to additional refinements prior to the launch.

### Search Log Analysis

**Figure 4:**
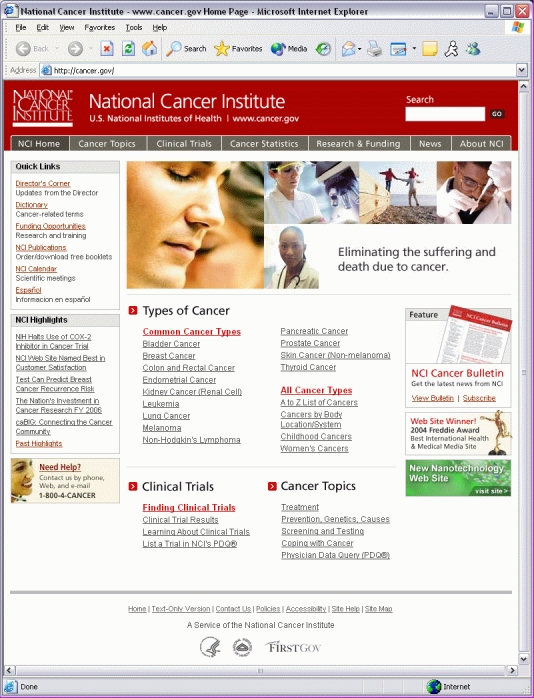
The home page of the current NCI website, prominently featuring “Types of Cancer” and links to “Common Cancer Types”

Search log analysis played an important role in the 2004 redesign of the NCI website. Each year, users enter approximately 2.5 million free-text searches in the basic search box on the site. More than 50% of searches are for types of cancer or specific body systems or locations. To give visitors immediate access to information on the most common cancers, prominent links for each of these cancers were added to the site's home page, along with multiple links to an A to Z list of cancers to enable easy information retrieval (Figure 4). The same selection of links to common cancers and the A to Z list was also placed on the site's Cancer Topics portal page (which replaced the Cancer Information portal page introduced in the 2002 redesign). (For more information about search log analysis, see “Best Bets on the Website” below.)

## Selected User-Driven Enhancements

### Best Bets on the Website

When the NCI website was redesigned in 2002, the site's search tool was supplemented with a “Best Bets” feature that gives users a concise list of editorially selected NCI sites and pages that are displayed above the full set of search results. Whereas the full set of search results, which are generated by a free-text search of NCI's Web content, can number thousands of documents for a given search term, the Best Bets offer an average of two links, with a range of one to 18. There are currently 677 Best Bets categories (eg, lung cancer, mammography, cancer diagnosis program) with selected Spanish-language categories included.

To populate Best Bets initially, a team of information experts identified cancer-related information categories, selected the most relevant NCI sites and pages for each, and created a table of related terms for each category name. When a search term is entered in the search box on the site and a category name or related term matches the term or any part of the term, the associated list of Best Bets is displayed.

Search log analysis after the launch of the Best Bets feature in 2002 validated the choice of category names and related terms, the majority of which proved to be among the more popular search terms. Since 2002, the Best Bets database has been edited by NCI staff as needed, on the basis of periodic analysis of search logs and knowledge of new and changing NCI Web content. Log analysis has prompted a considerable expansion of the Best Bets database by suggesting new category names and related terms. In addition, there have been a few instances in which the large number of searches on a topic indicated the need for new content. These findings have already led to the creation of two important pieces of content (which, in turn, were classified as Best Bets), namely a fact sheet about cancer staging and a substantial resource on the NCI website called the “Tobacco and Cancer” home page. Best Bets categories, related terms, and links have also been added in response to comments from users.

### Clinical Trials Portal Redesign

**Figure 5:**
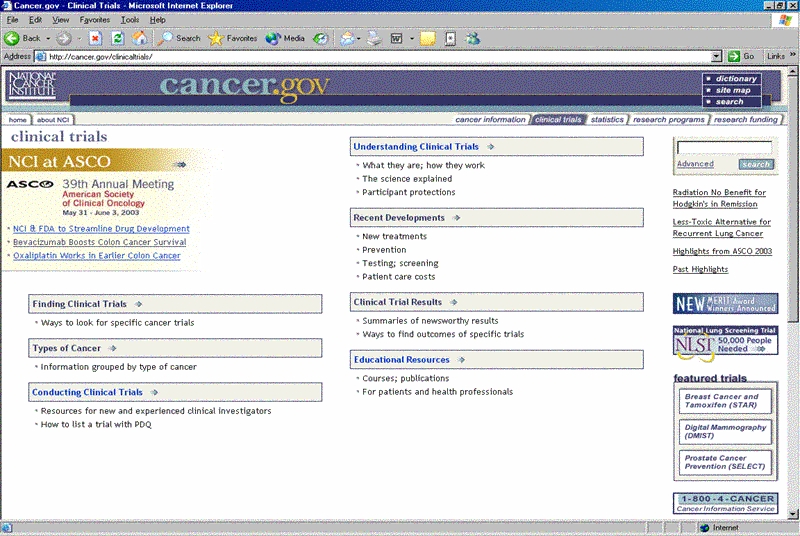
The Clinical Trials portal home page on NCI's website (2002)

In the summer and fall of 2002, NCI initiated a multi-pronged review of the Clinical Trials portal of its website (Figure 5) to determine whether the portal was meeting the needs of its users. Particular attention was given to the Clinical Trial Results section of the portal; articles in the section are also referred to as “news summaries” [13].

#### Evaluation Methods

The 2002 evaluation used six qualitative and quantitative methods:

1. The initial phase of the evaluation involved *key informant interviews* with NCI staff integral to the development of the portal.
2. A *diary activity* was conducted to capture feedback from users who were representative of three of the portal's target audiences, including patient advocates, oncology nurses, and CIS information specialists. Participants were asked to complete a written, formatted diary entry for each visit they made to the portal in the course of their regular activities over a period of one month.
3. *Focus groups* and *in-depth interviews* were later conducted to gain more feedback.
4. Two *online surveys* were posted in the Clinical Trials portal of NCI's website. A general survey was presented to each user who visited any page of the portal except for news summaries. A news summary survey was presented to users who visited news summaries. Session cookies were used to recognize possible repeat visitors and to serve up the survey once per visitor during a 30-minute time period (to minimize both the burden on the public and duplicate responses). Once duplicate responses were eliminated, the adjusted survey sample contained 1589 general survey responses and 207 news summary survey responses.
5. *Usability testing* was conducted to determine whether users could easily find and understand the news summaries. Perceived usefulness of the news summaries was also explored in usability testing with six participants.
6. Server log file entries were analyzed using WebTrends *log analysis* software to collect the following usage statistics: unique visitors, visitors who visited once, visitors who visited more than once, sessions, median visit length, page views, and visits from referring sites.

#### Key Findings

Several key findings emerged from these evaluation methods [14]. The top three categories of information that visitors were looking for were (1) specific cancer clinical trials (ie, they wanted to search the PDQ registry); (2) recent research results about a specific cancer treatment, test, or prevention; and (3) recent research about a specific type of cancer.

With regard to the Clinical Trials portal, most users found the information they needed, were able to understand it, and found it useful. However, they had difficulty finding their way to the Clinical Trial Results news summaries, even though this type of information was among the top three categories of information desired. When directed there (or when identified as having been there via the pop-up exit survey), users found the summaries to be useful, understandable, and well organized.

#### Informed Changes

**Figure 6:**
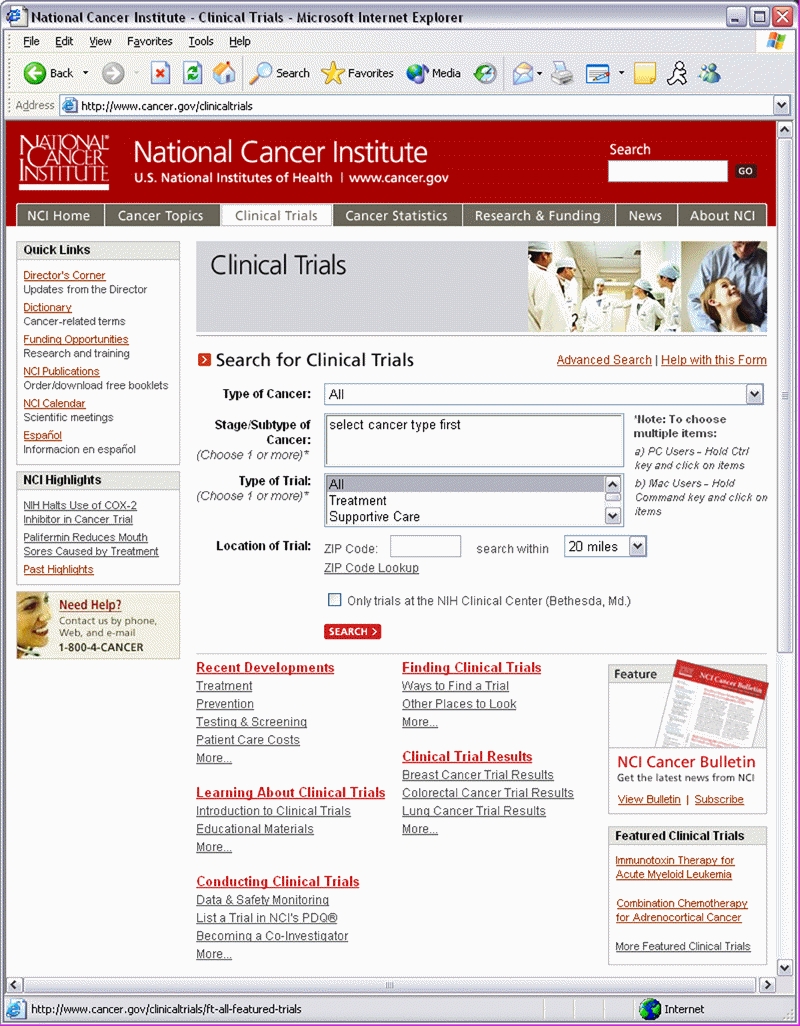
The Clinical Trials portal home page on the 2004 redesigned NCI website

These findings were used to inform changes to the content and design of the Clinical Trials portal over the course of 2003 and again during the 2004 redesign of the NCI website (Figure 6). For example, to make it quicker and easier for users to search the PDQ clinical trials registry, the website's basic search form for clinical trials was added to the Clinical Trials portal home page [15]. Links to this form are also located throughout the pages of the Clinical Trials portal and elsewhere on the site.

To further help visitors locate specific trials in which they might be interested, a new section was created called Featured Clinical Trials [16]. This section is updated on a weekly basis and includes brief profiles of key NCI-sponsored clinical trials, with links to more detailed information about the trial. Both the Featured Clinical Trials and the Clinical Trial Results sections were redesigned to allow users to browse by type of cancer and to search the collections by keyword from anywhere in the sections.

In addition, links to the Clinical Trial Results pages organized by type of cancer are more prominently displayed on the Clinical Trials portal home page, and teasers (brief description and link) for the two most recently posted Results articles are prominently displayed on the Clinical Trials portal home page.

### Improved Searching for Clinical Trials

The PDQ clinical trials registry has been a key component of NCI's online cancer information services from its inception in the 1980s [3]. Originally designed for health professionals, the registry is now also widely used by patients and is one of the most popular features of NCI's website. Since January 2003, more than 50000 visitors per month, on average, have searched for clinical trials. Designing a search application that works equally well for patients, caregivers, health professionals, and researchers has been a major challenge, and NCI has relied on feedback from users as well as insight from experts to guide each version of the clinical trials search form.

Since its appearance on the Web on CancerNet, the complexity of the clinical trials search form has been a topic of discussion within NCI. The PDQ clinical trials registry began as part of a DOS-based, menu-driven system used almost exclusively by health professionals, medical librarians, and cancer information specialists. Developers were wary of transplanting the sophisticated search functionality of this system to NCI's website because many of the site's visitors had little familiarity with clinical trials, cancer staging, treatment choices, and other elements in the original system. Initial methods of clinical trial searching on NCI's CancerNet website included a form with limited search options and clinical trial descriptions written in technical language, a legacy from the original system. Simply written, patient-friendly descriptions of clinical trials were introduced in 1997.

#### Two-Step Search Form

The second-generation search form that was launched in 1999 was based on recommendations from the Chantilly meeting, data from an online feedback form on the website, analysis of the search form, and personal interviews. In addition, a prototype of the form was developed through iterative rounds of usability testing. A two-step search form was designed to allow users to search by common search parameters, such as type of cancer, type of trial, and geographic location. Users could then review their search results or choose to narrow their search with other parameters, such as stage of cancer, drug (including brand and generic names), type of treatment, and trial sponsor. Users were also given the option of viewing two descriptions of each clinical trial, one for patients and one for health professionals.

Other changes to the search form based on usability testing included a user's guide for less experienced users, annotated labels for search parameters with links to more detailed explanations, and explanations of how to select multiple items per field (eg, selecting several stages of breast cancer).

#### Audience-Focused Search Form

In the 2002 redesign of the NCI website, the clinical trials search form was included in the Clinical Trials portal of the site, giving users a more integrated information pathway that grouped information such as patient safety, informed consent, and insurance issues with the listing of clinical trials.

A major consideration in this redesign was the addition of a specific new group of users—information specialists from the CIS, whose duties include assisting patients, their families, and health professionals in identifying clinical trials of interest. Information specialists had previously used the DOS-based, menu-driven PDQ search system that allowed them to perform complex searches, review results, refine as needed, and then prepare an “information packet” that could be emailed or mailed to callers. Web designers visited a CIS regional office to understand the needs of this group of users and did extensive usability testing with them prior to launching the revised form.

Given the diversity of users, it became clear that a single search form was not ideal. Some users found the detailed choices on the form confusing and beyond what they needed. An interactive search form that guided users through the search process was considered, but such a form would require JavaScript, which does not meet Web accessibility requirements for federal government websites. It was determined that the best approach was to develop two search forms with different levels of complexity. Web accessibility requirements could be satisfied by creating one form without JavaScript, and a more complex, interactive form could be created with JavaScript.

The basic search form, designed for the patient, caregiver, or busy health professional, provided three search options—cancer type, type of trial, and zip code proximity. The results were also presented in a format more suited to the casual Web user, who was accustomed to clicking on a search result link to go to a page that contained more information. Usability testing had also indicated that users did not normally click on the check boxes that were provided with the search results in order to prepare a “package” for viewing or printing as a batch.

The advanced search form [17] was JavaScript enabled with key enhancements that included (1) dynamic population of the cancer subtype/stage search options based on cancer type selection, and (2) expanded trial site and location searching, including searches by zip code proximity and hospital. In addition, browse lists for drugs, hospitals, and investigators were added to support more precise searching. Users could search for a character string and find appropriate values to add to the search form, or they could browse data-generated pick-lists alphabetically for drug, hospital, or investigator.

In addition, for the CIS users, the search results display was developed to enable information specialists to read a preliminary result set, so they could identify the most appropriate trials for their callers and prepare an “information packet.”

#### Better Visibility for Search Forms

The 2004 redesign of the NCI website saw further changes in clinical trials searching. Based on user input, the ability to narrow a search to subtype or stage of cancer was added to the basic search form. User feedback also indicated that physicians preferred trials to be listed by phase rather than by title, so the default display of search results was changed to a listing by phase, with phase IV and phase III trials appearing before phase II and phase I trials. The most substantial change, as a result of the Clinical Trials portal review, was adding the basic search form to the top of the Clinical Trials portal home page to give more ready access to the form [15]. With continued feedback from users, the search forms will be improved further to allow more precise clinical trial searching—for example, an interactive format may be developed to help identify trials with eligibility criteria that match patient characteristics.

### Patient-Oriented Clinical Trial Abstracts

In the summer of 1996, NCI collaborated with the National Alliance of Breast Cancer Organizations (NABCO) to develop patient-oriented abstracts (summaries) of clinical trial protocols for breast cancer trials. By October 1996, these clinical trial abstracts were available on the NABCO and CancerNet websites in a one-paragraph format. After seeking input from many advocacy organizations, the patient-oriented clinical trial abstract format was redesigned, writing guidelines were developed, and the project was expanded to include all cancer types. By September 1998, patient-oriented abstracts for all active clinical trials were available on CancerNet. Since that time, clinical trial abstracts have been written according to the original guidelines.

In November 2001, selected patient-oriented and corresponding health professional clinical trial abstracts were evaluated. As a result of this evaluation, several problems were identified in the guidelines for writing the patient-oriented abstracts, including a lack of specificity in some respects and inconsistent application and interpretation of the guidelines. These findings led to the recommendation that the guidelines be redefined and expanded. Consequently, a qualitative and quantitative evaluation of the needs and preferences of users of the patient-oriented abstracts was undertaken. This evaluation included the following two elements: (1) a written survey of advocacy organizations, members of NCI's Consumer Advocates in Research and Related Activities (CARRA) Program, members of the NCI Director's Consumer Liaison Group (DCLG), comprehensive cancer center directors and administrators, cancer cooperative group chairs/administrators, and oncology nurses; and (2) in-depth interviews with CIS information specialists.

#### Written Survey

A 10-question survey was mailed to nearly 400 organizations and individuals, with a 43% return rate. A key question focused on whether or not users could understand and act on the information provided in the clinical trial abstracts for patients. Results showed that 82% of users could explain the rationale or purpose of the clinical trial, 93% could determine if basic eligibility requirements were met, and 73% could understand the treatment plan.

The organization and layout of the clinical trial abstracts were rated “excellent” or “good” by 72% of the respondents. Three samples of text written at 5th-, 8th-, and 12th-grade reading levels were included with the survey. The different reading levels were preferred by 37%, 42%, and 20% of the respondents, respectively.

The results of the survey were better understood when viewed in the context of comments from individual respondents. Taken as a whole, the respondents' comments were varied and, at times, contradictory. Several themes, however, emerged related to language and readability, access to other resources, and pursuing participation in a clinical trial. Although 27% of the respondents indicated they could not understand the treatment plan, few specific suggestions were offered for improvement.

#### Interviews with CIS Information Specialists

Structured interviews were conducted with staff in six CIS offices in different geographic areas of the United States in order to obtain their perceptions of users' needs, preferences, and comprehension of the standard elements (title, rationale, purpose, eligibility criteria, treatment, and study contacts) of the patient-oriented clinical trial abstracts. The CIS information specialists interact directly with users of the abstracts by answering their questions and by guiding their use of the abstracts online during a phone call or through LiveHelp. The information specialists emphasized the need to use consumer-oriented language and the fact that users “skip” disclaimer-type information.

Based on these findings and on published principles [18], improvements to the patient-oriented abstracts were implemented as part of the 2004 redesign of the NCI website (Table 3). An example of the current abstract format [19] can be viewed online.

**Table 3:** Selected improvements to the patient-oriented clinical trial abstracts

**Criteria**	**Improvement**
Use of Language	Provide both simplified and health professional versions of the title. Avoid technical terms if a more common term is available (eg, “removed in surgery” instead of “resected”). Aim for an 8th-grade reading level or lower, except for drug names and medical or scientific terms defined in the website's dictionary (terms are linked to dictionary definitions).
Readability	Write sentences that are as short as the content will allow. Divide lengthy treatment descriptions into smaller paragraphs. Use bullets to separate information about different treatments.
Content Display	Emphasize how users who are interested in participating in a clinical trial can seek further information. Incorporate disclaimer information into the eligibility and trial contact information sections. Provide a boxed sidebar containing links to complementary information about clinical trials and drug information in the National Library of Medicine's MedlinePlus. Keywords in the title should not be linked to dictionary definitions. They should be linked from the purpose or treatment sections rather than the title.

### Web-Friendly Cancer Information Summaries for Patients

The PDQ cancer information summaries are descriptions of the latest cancer information on treatment, supportive care, screening, prevention, genetics, and complementary and alternative medicine that are reviewed and updated monthly by cancer experts. Most of the summaries are available in two versions: one written for health professionals and a corresponding patient version written in lay language. (A small number of the summaries are available only in the health professional version.) In 2000, in response to the Chantilly meeting, work was initiated to reformat the patient-oriented information summaries. The goal was to present the information in a format and style of language that was easier to read and understand, to provide more detailed information, and to take advantage of features afforded by new Web technology.

Based on design concepts that enhance readability, as well as on strategies used in information mapping, the process of reformatting and reorganizing the patient-oriented summaries was begun. “Key Points” boxes that highlighted critical concepts and linked to explanatory information in the body text were added. Links to pop-up definitions from the website's dictionary and to clinical trials information were included. For users who wished to print documents, a printer-friendly version was added that included dictionary terms and their definitions as an appended glossary.

Usability testing was done to assess the ease of learning, efficiency in information gathering, and recall of information from the online documents. Based on testing results and Web design and usability guidelines [18], the template for the patient-oriented summaries was further refined, and the redesign has been well received by users. An example of the current summary format [20] can be viewed online.

## Conclusion

NCI's website is a leading resource for cancer information on the Web, consistently appearing high on the list of retrievals using search engines such as Google, Yahoo, MSN, and AltaVista. It has been awarded the Freddie Award in the website category of the 2004 International Health and Medical Media Awards, and it placed first or as an honorable mention in seven out of eight categories in the 2005 Medicine on the Net Web Excellence Awards. Its success can be at least partly attributed to NCI's efforts to make the site highly responsive to the needs of its users.

The large volume of traffic that the site receives offers tremendous opportunities to study user patterns, gather feedback, and test new ideas and designs. Online surveys are an efficient way to solicit opinions from users, and analysis of website logs provides insight into user needs. NCI's relationships with members of the cancer research and advocacy communities also facilitate the gathering of advice, suggestions, and other feedback related to NCI information products. The growing body of Web-design literature and advice from usability experts are important to the development of new Web features, but input from the site's wide range of users promises to have the greatest impact on shaping online information from the National Cancer Institute.

## Appendix 1

### American Customer Satisfaction Index (ACSI) Survey

The following questions are from the American Customer Satisfaction Index (ACSI) survey currently used on all pages of the NCI website (except pages selected for a customized survey). In this pop-up survey, each of the first 16 questions is followed by a numbered scale (1 = poor, 10 = excellent; or 1 = not very likely, 10 = very likely), and the final 12 questions are followed by pull-down menus, a field for typing text, or lists of choices with check boxes or radio buttons. (Copyright 2004 by ForeSee Results)

1. Please rate the **accuracy of information** on this site.
2. Please rate the **freshness of content** on this site.
3. Please rate the **usefulness of the information provided** on this site.
4. Please rate the **ability to accomplish what you wanted to** on this site.
5. Please rate the **ease of navigation** on this site.
6. Please rate how this site **provides comprehensive search results**.
7. Please rate the **organization of search results** for this site.
8. Please rate the **speed of loading the page** on this site.
9. Please rate the **consistency of speed** on this site.
10. Please rate the **reliability of site performance** on this site.
11. What is your **overall satisfaction** with this site?
12. How well does this site **meet your expectations**?
13. How does this site **compare to your idea of an ideal website**?
14. How likely are you to **return to this site**?
15. How likely are you to **recommend this site to someone else**?
16. How likely are you to use this site as your **primary resource**?
17. How **frequently** do you visit this site?
18. Which of the following best describes your **role** in coming to Cancer.gov?
19. If you answered “Other” for your role, please specify.
20. Please complete this sentence: I am visiting Cancer.gov today to find information on ______.
21. If you answered “Other” or “Other cancer-related information” for why you are visiting this site, please specify.
22. Please rate how much you agree or disagree with the following statement: The information I found on this site was too hard to understand.
23. Which of the following best describes the highest level of education you have completed?
24. What is your **gender**?
25. How do you describe your **ethnicity**?
26. How do you describe your **race**?
27. Please select the category that includes your age.
28. If you could make one improvement to this site, what would it be?

## Appendix 2

### Sample Scenarios for Usability Testing of the NCI Website

1. Can you find NCI press releases about breast implants?
2. You want to know if NCI will fund research on tobacco control for ethnic populations. Where would you look?
3. Where can you find the policies for protecting people who participate in clinical research studies?
4. Where can you find an online (electronic) publication that explains radiation therapy?
5. You'd like information about Hodgkin's disease. What can you find?
6. Where can you find a list of the NCI's clinical research labs/branches in Bethesda, Maryland?
7. Where would you look to find clinical research results reported at scientific meetings?
8. There was a news story about a drug called cyproterone acetate, used to reduce hot flashes following surgery for prostate cancer. You want to know if a man with prostate cancer in Augusta, Georgia, can enroll in a clinical trial that uses this drug.
9. You are looking for a list of phase II melanoma trials that use vaccine therapy and are being conducted at the NIH.
10. A women's group is planning a breast cancer awareness seminar and would like a list of breast cancer screening and prevention studies in their 07112 ZIP Code.
11. You are looking for information about a trial called CLB-49907.
12. There was a newspaper article about a physician, Dr. Tanya Trippett, at Memorial Sloan-Kettering in New York, who is conducting a breast cancer trial. You don't remember the name of the study but would like to get in touch with Dr. Trippett.

## Abbreviations

ACSI: American Customer Satisfaction Index
CIS: Cancer Information Service
NCI: National Cancer Institute
PDQ: Physician Data Query database
